# Addendum: Implicit learning of temporal behavior in complex dynamic environments

**DOI:** 10.3758/s13423-022-02194-x

**Published:** 2022-10-17

**Authors:** Josh M. Salet, Nadine Schlichting, Wouter Kruijne, Hedderik van Rijn

**Affiliations:** 1 Department of Experimental Psychology, University of Groningen, Groningen, Netherlands; 2 Institute for Experimental Psychology, Heinrich Heine University Düsseldorf, Düsseldorf, Germany

**Keywords:** Temporal preparation, Statistical learning, Motor planning, Time perception

## Abstract

New analyses of the data in this study (Salet et al., 2021, *Psychonomic Bulletin & Review,* 10.3758/s13423-020-01873-x) have led us to reinterpret our main finding. Previously, we had attributed better performance for targets appearing at regular intervals versus irregular intervals to “temporal statistical learning.” That is, we surmised that this benefit for the regular intervals arises because participants implicitly distilled the regular 3000 ms interval from the otherwise variable environment (i.e., irregular intervals) to predict future (regular) targets. The analyses presented in this Addendum, however, show that this benefit can be attributed to ongoing “temporal preparation” rather than temporal statistical learning.

## Method and results

Details about the experiment and statistical methods are reported in Salet et al. (2021). Here, we report the reanalyses of Experiment 1 as a high-level overview. The reanalysis of Experiment 2 results in similar conclusions and is reported in the Appendix to this Addendum. All analyses are described in more detail on our OSF repository (https://osf.io/9fp43/).

## Experiment 1

### Temporal statistical learning

To briefly summarize, our main empirical finding (Figs. 2 and 3 in Salet et al., 2021) was that reaction time (RT) was lower and hit rate (HR) was higher for regular targets compared to irregular targets (replicated in Fig. 1a and c). Critically, we found this to be the case even in the implicit phase, in which participants were unaware of the regularity. We concluded that “participants adapted to the temporal regularity without detecting its presence, and thus without intentionally utilizing temporal information” (Salet et al., 2021, p. 8). This interpretation could reflect a case of temporal statistical learning showing that participants, outside their awareness, automatically adapt their behavior to the statistical regularities in the environment.

**Fig. 1:**
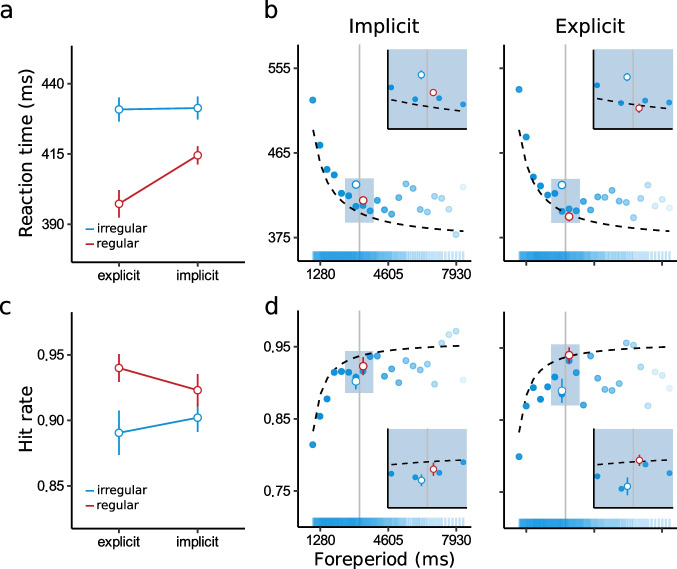
Temporal preparation in Experiment 1. Average (**a**) reaction time (RT) and (**c**) hit rate (HR) for the implicit and explicit phases (as in Salet et al., 2021). Temporal preparation: (**b**) mean RT decreases and (**d**) HR increases as a function of foreperiod (FP), characteristic of temporal preparation effects for both regular and irregular targets. The data points represent binned RT and HR (bin size = 350 ms), the dashed lines, the fit of the statistical model. For illustration purposes, we cut off 1% of the tail of the data (FP > 8,000 ms). Opacity of the colored bar above the *x*-axis codes for the amount of data per FP bin. Solid vertical line represents the regular FP of 3,000 ms. (Color figure online)

### Temporal preparation

As the timing of irregular target onsets was highly variable (ranging from 750 to 18,000 ms), we had originally considered these to be unpredictable and unlikely to be timed. However, in our follow-up work Salet et al. (2022), we have come to realize that the temporal information embedded in the stream of irregular targets has a marked effect on behavior. A ubiquitous finding in studies on temporal preparation is that in blocks with variable preparation intervals, longer intervals lead to faster responses (Nobre & van Ede, 2018; Salet et al., 2022). For example, an irregular target that has not appeared for a long time (e.g., > 3,000 ms) may be expected to come on soon and, as a consequence of preparation, result in a speeded response.

### Preparing irregular targets

To assess whether participants indeed prepared for irregular targets, we evaluated whether we could observe a decrease in RT and increase in HR as a function of the interval between subsequent irregular targets. From here on, we refer to this interval as the foreperiod (FP, illustrated in Fig. 2), in line with the terminology used in temporal preparation research. This FP was, by definition, 3,000 ms for regular targets (Fig. 2, red row). For the other targets the interval ranged from 750 to 18,000 ms with a FP distribution as indicated in Fig. 1b and d (see colored bar on the *x*-axis).

**Fig. 2:**
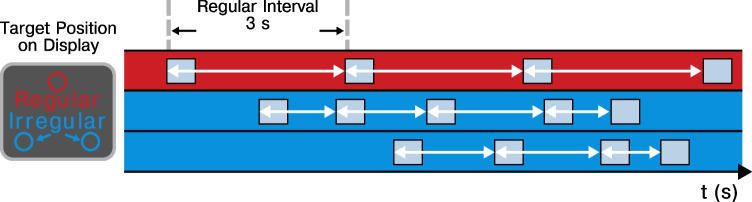
Foreperiods. Segment of the sequence illustrating the foreperiods (FPs). The red row represents the timing of the regular target (constant FP of 3,000 ms), the blue rows the irregular targets (FPs ranging from 750–18,000 ms). Figure adapted from Salet et al. (2021). (Color figure online)

To test for preparation effects, we first took the best “2021 model” without FP of both RT and HR reported in Salet et al. (2021). To recap, for RT, the best 2021 model included “regularity” (regular versus irregular), “phase” (explicit versus implicit), and their interaction as predictors. For HR, only “regularity.” We then created a new model in which we added FP as a continuous predictor and its interaction with “phase” to the best 2021 model. We refer to this new model as the “addendum model.” Of note, we use 1/FP as a predictor in order to better capture the curvilinear nature of this relationship (Fig. 1b and d). This vastly improved the models of both RT and HR compared with a linear relationship (ΔBIC > 147, BF > 1,000).

### Is temporal statistical learning an illusion?

Model comparisons revealed the best addendum model of both RT and HR to include only FP as predictor. Critically, we found the addendum model to vastly improve the fit compared with the 2021 model (ΔBIC > 85, BF > 1,000), providing strong evidence for the absence of an effect of regularity. This evidence against a role of regularity illustrates that the observed adaptation to the regular target (Salet et al., 2021) can be seen as an artifact of temporal preparation effects. This is illustrated in Fig. 1b and d where we depict the average RT and HR as a function of regularity (as reported in Figs. 2 and 3 of Salet et al., 2021) and FP. It can be seen that, for irregular targets, short FPs (i.e., < 3,000 ms) are characterized by high RTs and low HRs. Due to the strong asymptotic nature of preparation effects, long FPs (> 3,000 ms) are not affected to the same extent. This asymmetry leads to an apparent difference for regular and irregular targets when only considering the average RT and HR as a function of regularity (as in Salet et al., 2021). However, when observing performance as a function of FP, it can be seen that at FP = 3,000 ms (Fig. 1b and d), there is no apparent difference between regular and irregular intervals.

## Discussion

These new analyses show that participants implicitly make use of the temporal information embedded in the irregular stimulus sequences. That is, RT decreased and HR increased as a function of FP; in a manner that is characteristic of temporal preparation (Los et al., 2014; Nobre & van Ede, 2018; Salet et al., 2022). Importantly, these findings call for a reinterpretation of the main effects in Salet et al. (2021): That is, better performance for the regularity does not reflect temporal statistical learning, but instead is due to temporal preparation. As illustrated in Fig. 1, temporal preparation drives the average effects across FPs (Fig. 1, intercept plots). Solely observing the average effects split on regularity (as in Salet et al., 2021) thus provides the illusion of temporal statistical learning of the regular interval.

### Conclusion

This Addendum shows that the effects we have previously attributed to temporal statistical learning are better explained as a consequence of temporal preparation: Better average performance for the regular compared with irregular intervals does not reflect a statistical learning mechanism that distills regular events from a fuzzy environment to predict future (regular) events. Instead, our analyses show that this average difference naturally arises from the consequences of a more continuous pattern of temporal preparation.

## Figures and Tables

**Fig. 3:**
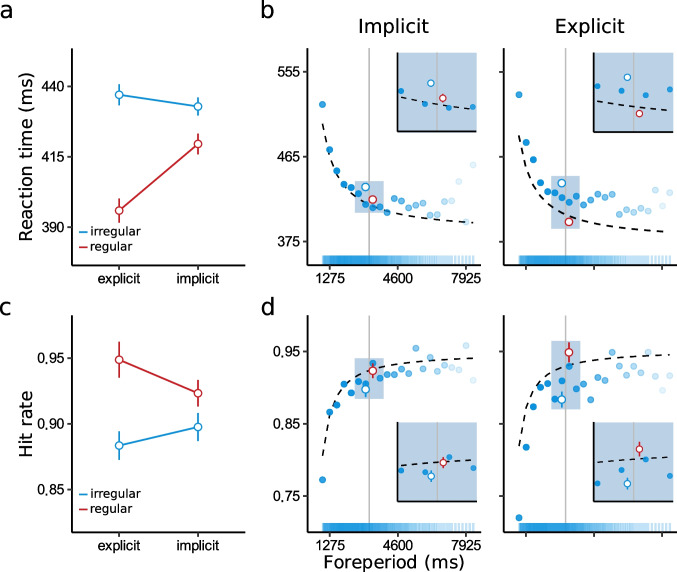
Temporal preparation in Experiment 2. Plotted as in Fig. 1 of the main text

## Appendix

### Reanalysis of Experiment 2

Here, we report the outcome of the reanalyses of Experiment 2, which led to similar conclusions as in Experiment 1 (Appendix Fig. 3).

### Anticipating irregular targets

As in Experiment 1, we created a new addendum model by adding FP as a continuous predictor and its interaction with “phase” to the best 2021 model reported in Salet et al. (2021). The best 2021 model included the predictor “regularity” (regular versus irregular), “phase” (explicit versus implicit), and their interaction, both for RT and HR.

### Temporal statistical learning an illusion?

Again, we found the best addendum model of both RT and HR to improve the fit compared to the 2021 model (ΔBIC > 28, BF > 1000).

In contrast to Experiment 1 (main text), the addendum model included the interaction between ‘phase’ and ‘regularity’. Importantly, however, the outcome of the post hoc Tukey’s HSD test of this interaction was in stark contrast with our 2021 report (Salet et al. 2021), revealing better performance for the regularity only in the explicit phase (RT: *z* = 8.76, *p* < 0.001; HR: *z* = -10.7, *p* < 0.001), but not in the implicit phase (RT: *z* = 1.3, *p* = 0.210; HR: *z* = -1.67, *p* = 0.095). This was further supported by Bayesian model comparisons of models separately fitted to data from the explicit (ΔBIC > 35, BF > 1000 in favor of a regularity effect) and the implicit phase (ΔBIC > 4, BF > 7 against a regularity effect).

These results indicate, for Experiment 2, an effect of regularity on top of preparation, although this was limited to the explicit phase. This effect is illustrated in Appendix Figure 3b and d. In the explicit phase, performance was better for regular versus irregular targets, even when accounting for temporal preparation (FP = 3000 ms in Appendix Figure 3b and d). This might suggest that participants, when explicitly instructed, keep track of the 3000 ms regular interval and use it to predict when to act, while at the same time preparing for irregular targets. Alternatively, instead of tracking the 3000 ms interval, explicit knowledge about the regularity might have led participants to prioritize this regular target over irregular targets, in a manner independent of their timing. Of note, we did not find an effect of regularity in the explicit phase of Experiment 1. However, indications for a similar trend are present in Appendix Figure 3b and d (main text).
